# Neuropsychiatric Outcomes of IL‐17 and IL‐23 Inhibitors in Dermatology: Risks, Benefits, and Clinical Implications

**DOI:** 10.1111/ijd.70204

**Published:** 2025-12-19

**Authors:** Isabella J. Tan, Alexis Mitelman, Mohammad Jafferany

**Affiliations:** ^1^ Rutgers Robert Wood Johnson Medical School New Brunswick New Jersey USA; ^2^ Weill Cornell Medical College New York New York USA; ^3^ Department of Psychiatry and Behavioral Sciences Central Michigan University College of Medicine Mount Pleasant Michigan USA

**Keywords:** depression, IL‐17 inhibitors, IL‐23 inhibitors, neuropsychiatric adverse events, psoriasis, suicidality

Dear Editor,

1

Interleukin (IL)‐17 and IL‐23 inhibitors have transformed the management of chronic inflammatory dermatoses, including psoriasis and hidradenitis suppurativa (HS), offering rapid skin clearance, reduced systemic inflammation, and improved patient‐reported quality of life [1]. Beyond cutaneous benefits, systemic inflammation contributes to fatigue, sleep disturbance, cognitive symptoms, and mood dysregulation, raising questions about whether cytokine‐targeted therapies modulate neuropsychiatric outcomes.

Randomized trials in psoriasis demonstrate low rates of depression and suicidality with IL‐17 and IL‐23 blockade (Table 1) [2]. For secukinumab and ixekizumab, reported depression ranges 0.2%–0.7%, with suicidal ideation rare and comparable to placebo [2]. Brodalumab carries a United States Food and Drug Administration (FDA) boxed warning due to rare completed suicides, predominantly in patients with prior psychiatric history, underscoring the importance of baseline risk assessment [2]. IL‐23 inhibitors (guselkumab, risankizumab, tildrakizumab) consistently demonstrate minimal neuropsychiatric risk, with depression in 0.2%–0.5% and no suicidal events reported [2].

**TABLE 1:** Neuropsychiatric outcomes reported with IL‐17 and IL‐23 inhibitors in dermatology.

Agent	Target	Pivotal trials (duration)	Reported neuropsychiatric outcomes (from pivotal trials unless otherwise specified)	Boxed warning/signal	Real‐world/registry data	Interpretation
Secukinumab	IL‐17A	ERASURE, FIXTURE; LTE ≤ 5 years	Depression 0.2%–1.0%; suicidality not elevated vs. placebo	None	Registry/claims: low incidence; improvements in mood often parallel skin clearance (follow‐up ≤ 5 years)	Favorable profile; no clear signal
Ixekizumab	IL‐17A	UNCOVER‐1/2/3; LTE > 3 years	Depression 0.3%–0.7%; rare suicidal ideation (< 0.1%), not > placebo	None	Real‐world data confirm low risk (up to 3 years)	Similar to placebo; generally safe
Brodalumab	IL‐17RA	AMAGINE trials; LTE ≤ 5 years	4 suicides in trials (3840 pts); suicidal ideation/events reported, often in pts. with psychiatric history	**FDA boxed warning for suicidal ideation/behavior**	FAERS: elevated reporting ratio for suicidality vs. other IL‐17 s (registry follow‐up ≤ 5 years)	Requires vigilant psychiatric monitoring; risk–benefit individualized
Bimekizumab	IL‐17A/F	BE‐READY, BE‐VIVID; LTE ≥ 3 years	Depression/anxiety < 1%; no suicides; emerging evidence of mood improvement	None	Post‐marketing: low incidence (follow‐up ≈3 years)	Possible protective effect; data maturing
Guselkumab	IL‐23p19	VOYAGE, NAVIGATE; LTE ≤ 5 years	Depression 0.2%–0.5%; anxiety < 0.5%; no suicidality	None	Registry/claims: low risk; QoL/mood improved with clearance (up to 4 years)	Consistently favorable profile
Tildrakizumab	IL‐23p19	reSURFACE; LTE ≤ 5 years	Rare depression/anxiety (< 0.5%); no suicidality	None	Sparse real‐world data; no signal (follow‐up ≤ 5 years)	Neutral to favorable
Risankizumab	IL‐23p19	UltIMMa, IMMhance; LTE ≤ 5 years	Depression 0.2%–0.5%; no suicidality	None	Registry data: low risk (follow‐up ≤ 5 years)	Favorable tolerability; lowest reported neuropsychiatric event rate among IL‐23 inhibitors to date, though no head‐to‐head trials confirm class differences

Emerging data in HS, a population with high baseline psychiatric comorbidity, suggest IL‐17 inhibition improves disease activity and may indirectly enhance mood [3]. Observational studies and registry data show improvements in fatigue, sleep, and depressive symptoms paralleling disease control [4]. Real‐world pharmacovigilance corroborates randomized controlled trial (RCT) findings: most patients tolerate therapy well, with rare paradoxical depressive events concentrated in those with pre‐existing psychiatric vulnerability [4].

Mechanistically, IL‐17 and IL‐23 modulate neuroimmune signaling. IL‐17A crosses the blood–brain barrier under inflammatory conditions, activating microglia, disrupting hippocampal synaptic plasticity, and impairing glutamatergic neurotransmission [5]. IL‐23 sustains Th17 differentiation and amplifies IL‐17–driven CNS effects. Cytokine blockade may confer indirect antidepressant effects through reduced systemic and neuroinflammation, while rare paradoxical events may emerge in susceptible patients [5]. Proposed mechanisms include maladaptive immune rebound or altered central nervous system (CNS) cytokine tone following abrupt cytokine blockade. In susceptible individuals, rapid downregulation of peripheral IL‐17/IL‐23 signaling may transiently unmask pro‐inflammatory cascades in the CNS, particularly in the context of pre‐existing neuroinflammation or dysregulated hypothalamic‐pituitary‐adrenal (HPA)‐axis activity [5]. These paradoxical events likely reflect an idiosyncratic neuroimmune disequilibrium rather than a direct drug effect. Skin clearance itself reduces peripheral cytokines and psychosocial stress, contributing further to mood improvement.

Adolescents, young adults, and patients with baseline depression, anxiety, or chronic pain syndromes represent higher‐risk subgroups. Structured baseline assessment (PHQ‐9, GAD‐7, C‐SSRS) is recommended, with longitudinal monitoring during therapy (Table 2). Clinical vigilance is particularly warranted with brodalumab, whereas IL‐23 inhibitors and IL‐17 inhibitors excluding brodalumab demonstrate favorable neuropsychiatric profiles [3].

**TABLE 2:** Proposed psychiatric screening algorithm before and during IL‐17/23 inhibitor use.

Timepoint	Recommended actions	Tools/metrics	Clinical triggers for escalation
Baseline (pre‐treatment initiation)	Obtain psychiatric history (depression, anxiety, suicidality, past psychiatric hospitalizations, current medications) Screen all patients using validated tools Educate patients on potential psychiatric symptoms and encourage prompt reporting Consider psychiatry referral in high‐risk individuals (active depression, prior suicide attempt, severe anxiety)	PHQ‐9 (Patient Health Questionnaire‐9) GAD‐7 (Generalized Anxiety Disorder‐7) Columbia Suicide Severity Rating Scale (C‐SSRS), if indicated	PHQ‐9 ≥ 10 or GAD‐7 ≥ 10 (moderate severity) Any suicidal ideation (PHQ‐9 item 9 > 0 or positive C‐SSRS) Recent suicide attempt or active severe psychiatric disorder
Early treatment (first 3 months)	Reassess mood at each visit (in person or telehealth) Monitor for new or worsening depressive symptoms, anxiety, sleep disturbance, fatigue, irritability, or suicidal thoughts Reinforce patient self‐monitoring and reporting	Repeat PHQ‐9 and GAD‐7 at 4–12 weeks Clinical interview	New onset suicidal ideation PHQ‐9 increase ≥ 5 points from baseline Any functional impairment linked to mood symptoms
Maintenance (every 6–12 months, or more frequently in high‐risk patients)	Continue structured screening at each visit Collaborate with psychiatry or primary care if psychiatric symptoms persist or worsen	PHQ‐9 and GAD‐7 every 6–12 months C‐SSRS if suicidality suspected	Worsening depression or anxiety scores Emergent suicidality Patient/family report of behavioral change
High‐risk scenarios (brodalumab initiation, adolescents/young adults, strong psychiatric history)	Require closer monitoring (every 4–8 weeks during the first 6 months) Pre‐emptive psychiatry referral strongly recommended Document detailed informed consent regarding neuropsychiatric risk	PHQ‐9, GAD‐7, C‐SSRS at each follow‐up during first 6 months	Any suicidal ideation or attempt Severe depressive or anxiety symptoms Inability to ensure safety or adherence

Indirect effects of biologics on fatigue, sleep, and cognition are increasingly recognized. Observational data show Psoriasis Area and Severity Index (PASI) 75–90 responders report improved energy, sleep quality, and mood, suggesting that systemic cytokine reduction and psychosocial benefits of visible skin improvement contribute meaningfully to mental health [3]. Integration of dermatologic and psychiatric care can optimize both safety and efficacy, particularly in HS and patients with comorbid psychiatric disease.

IL‐17 and IL‐23 inhibitors are generally safe regarding neuropsychiatric outcomes and may confer indirect mood benefits through systemic and cutaneous disease control. Rare adverse events, especially with brodalumab, highlight the need for careful baseline psychiatric evaluation and ongoing monitoring. Prospective studies with structured psychiatric endpoints, extended follow‐up, and integration of biomarker or neuroimaging correlates are needed to clarify CNS effects and guide personalized therapy. Embedding standardized neuropsychiatric assessments in dermatology trials will inform risk stratification and maximize the therapeutic potential of IL‐17/IL‐23 inhibitors.

## Funding

The authors have nothing to report.

## Conflicts of Interest

The authors declare no conflicts of interest.

## Data Availability

The data that support the findings of this study are available from the corresponding author upon reasonable request.

## References

[1] K. Knecht‐Gurwin , L. Matusiak , and J. C. Szepietowski , “The Preclinical Discovery and Development of Secukinumab for the Treatment of Moderate‐To‐Severe Hidradenitis Suppurativa,” Expert Opinion on Drug Discovery 20, no. 4 (2025): 405–417, 10.1080/17460441.2025.2482058. 40106842

[2] K. Ghoreschi , A. Balato , C. Enerbäck , and R. Sabat , “Therapeutics Targeting the IL‐23 and IL‐17 Pathway in Psoriasis,” Lancet 397, no. 10275 (2021): 754–766, 10.1016/S0140-6736(21)00184-7. 33515492

[3] M. Fabrazzo , S. Cipolla , S. Signoriello , et al., “A Systematic Review on Shared Biological Mechanisms of Depression and Anxiety in Comorbidity With Psoriasis, Atopic Dermatitis, and Hidradenitis Suppurativa,” European Psychiatry 64, no. 1 (2021): e71, 10.1192/j.eurpsy.2021.2249. 34819201 PMC8668448

[4] G. Pinto Salgueiro , O. Yilmaz , M. Nogueira , and T. Torres , “Interleukin‐17 Inhibitors in the Treatment of Hidradenitis Suppurativa,” BioDrugs 39, no. 1 (2025): 53–74, 10.1007/s40259-024-00687-w. 39604776 PMC11750882

[5] Y. Lu , P. Zhang , F. Xu , Y. Zheng , and H. Zhao , “Advances in the Study of IL‐17 in Neurological Diseases and Mental Disorders,” Frontiers in Neurology 14 (2023): 1284304, 10.3389/fneur.2023.1284304. 38046578 PMC10690603

